# Clinicopathological and Fecal Proteome Evaluations in 16 Dogs Presenting Chronic Diarrhea Associated with Lymphangiectasia

**DOI:** 10.3390/vetsci8100242

**Published:** 2021-10-19

**Authors:** Giacomo Rossi, Alessandra Gavazza, Silvia Vincenzetti, Sara Mangiaterra, Livio Galosi, Andrea Marchegiani, Graziano Pengo, Gianni Sagratini, Massimo Ricciutelli, Matteo Cerquetella

**Affiliations:** 1 School of Biosciences and Veterinary Medicine, University of Camerino, Via Circonvallazione 93/95, 62024 Matelica, Italy; giacomo.rossi@unicam.it (G.R.); alessandra.gavazza@unicam.it (A.G.); silvia.vincenzetti@unicam.it (S.V.); livio.galosi@unicam.it (L.G.); andrea.marchegiani@unicam.it (A.M.); matteo.cerquetella@unicam.it (M.C.); 2 St. Antonio Veterinary Clinic, S.S. 415 Paullese 6, 26020 Madignano, Italy; graziano@cvsantantonio.eu; 3 School of Pharmacy, University of Camerino, Via Sant’Agostino 1, 62032 Camerino, Italy; gianni.sagratini@unicam.it (G.S.); massimo.ricciutelli@unicam.it (M.R.)

**Keywords:** chronic diarrhea, diagnostic markers, dog, fecal proteomics, lymphangiectasia

## Abstract

Canine intestinal lymphangiectasia (IL) is a condition characterized by variably severe gastrointestinal signs, frequently associated with laboratory abnormalities; the research for markers allowing a better understanding of the severity degree and/or obtaining an early diagnosis and/or monitoring is continuously progressing. In the present study, we investigated possible new diagnostic/follow-up markers in IL dogs, namely, serum C-reactive protein, serum bacterial lipopolysaccharide, serum cleaved cytokeratin 18, serum citrulline, and zonulin (in both serum and feces). A fecal proteomic study looking for possible confirmation and/or new marker candidates was also performed. All markers in both substrates, with the exception of serum citrulline, significantly differed between diseased and control dogs. Fecal proteomics allowed the retrieval of three proteins in IL dogs (Fc fragment of IgG-binding protein; transthyretin; proproteinase E) that were not previously found in clinically healthy subjects. Although further studies are needed, C-reactive protein, bacterial lipopolysaccharide, cleaved cytokeratin 18, and zonulin (in both serum and feces) resulted as promising markers for canine IL; similarly, fecal proteomics represents a road worthy of being pursued in the search for candidate biomarkers.

## 1. Introduction

Canine intestinal lymphangiectasia (IL) is a condition characterized by variably severe gastrointestinal (GI) signs, frequently associated with laboratory abnormalities involving albumin and cholesterol levels [1]. It is histologically characterized by variable degrees of lacteal dilatation, possibly leading to protein dispersion and subsequent protein-losing enteropathy (PLE) [1,2,3], also justifying malabsorption and participating in the genesis of clinical signs. The condition can be idiopathic or associated with infiltrative inflammatory/neoplastic conditions causing a reduction in lymphatic drainage, such as inflammatory bowel disease (IBD) or venous hypertension [1,2,4,5,6,7].

IL can be diagnosed by performing histopathology on intestinal biopsy samples, with the primary form necessitating an exclusion diagnostic path to be differentiated from the secondary one [2,7,8]. However, the research for markers allowing (i) the exact understanding of the severity degree, and/or (ii) an early diagnosis, as well as generally predicting the histologic appearance or granting the follow-up of the disease, is ongoing [6]. Hypoalbuminemia, hypocholesterolemia, lymphopenia, and hypocalcemia have been associated with IL, while other possible markers such as α1-proteinase inhibitor (α1-PI), blood urea nitrogen (BUN), C-reactive protein (CRP), and packed cell volume (PCV) have also been investigated for the abovementioned purposes [2,6]; similarly, in the review by Craven et al. other molecules such as cobalamin, perinuclear antineutrophil cytoplasmic antibodies, S100A12 (calgranulin-C), and calprotectin were studied in dogs presenting protein-losing enteropathy [7]. Zonulin, one of the few known physiological mediators of paracellular intestinal permeability by modulating intercellular tight junctions (TJs) [9,10], is a molecule identified as pre-haptoglobin 2 (pre-HP2) [10]. The immunomodulatory hemoglobin-binding protein haptoglobin (HP) gene harbors a common polymorphism with two different alleles: *HP1* and *HP2*; in human, allele *HP2* (genotype *HP22*) has been shown to be overrepresented in different immune diseases, as well as in patients with IBD (ulcerative colitis—UC and Crohn’s disease—CD) [11,12,13,14], type-1-diabetes (T1D) and insulin resistance [15], irritable bowel syndrome (IBS) [16], necrotizing enterocolitis [17], and nonceliac gluten sensitivity [18], compared to healthy controls [19]. In human, zonulin increases intestinal permeability in the small intestine and participates in intestinal innate immunity. Actually, circulating zonulin in serum is considered a useful marker of intestinal permeability [10,20].

Fecal proteomics (FP) was recently introduced in veterinary medicine in both healthy dogs and healthy cats, as well as in dogs suffering from food-responsive diarrhea [21,22,23]. The study of the proteome of a certain body district involves an analysis of all the proteins present in it, in both physiological and pathological conditions, aiming at the possible discovery of new markers, allowing an earlier or more precise diagnosis of different diseases, while also permitting a better evaluation of the response to therapies or the onset of any relapses [22,23].

In the present study, we investigated new possible diagnostic/follow-up markers for IL using a double approach. After including dogs presenting chronic diarrhea, classifying them clinically by CIBDAI score [24], and performing intestinal histopathology confirming the lymphangiectasia (16 patients), we (i) investigated the potential of some molecules for that aim, namely, C-reactive protein (CRP), bacterial lipopolysaccharide (LPS) that can be considered an indirect indicator of proinflammatory activity, cleaved cytokeratin 18 (cCK18) as a marker of epithelial apoptosis, citrulline as an indicator of intestinal absorption, and zonulin (in both serum and feces) as a marker of the intestinal barrier integrity, comparing results from IL patients and the control group, and (ii) completed a fecal proteomic study looking for possible confirmation and/or new marker candidates.

## 2. Materials and Methods

### 2.1. Patients

In the present study, we included 16 dogs presenting chronic diarrhea (and variably presenting weight loss, steatorrhea, or malabsorption), with endoscopic and histological evidence of intestinal lymphangiectasia (IL Group—ILG), in the absence of other comorbidities [25]. Parallelly, seven dogs evaluated for other GI conditions (e.g., GI dysmotility, irritable bowel syndrome) and not diagnosed with IL were also included as controls (C group—CG). Only for zonulin (serum and feces), controls constituted 10 subjects, as three more were added. In the ILG, nine dogs were males and seven were females, while the mean age was 7.2 years (range 7–8); in the CG, four dogs were males and six were females, while the mean age was 5.2 years (range 3–8). Patients of both groups were scored clinically by CIBDAI score as previously reported. Copromicroscopic and *Giardia* evaluations were also performed, resulting negative, as inclusion criteria in the study.

### 2.2. Intestinal Markers

Raw stool samples from the ILG and CG groups were frozen and stored at −80 °C within 12 h of sampling. Before the laboratory analysis, stool samples were thawed, and mechanical homogenization was performed using an inoculation loop. The Fecal Sample Preparation kit (Roche Diagnostics, Germany) for the preparation of fecal eluates was used. In this system, 100 mg of stool sample was suspended in 5 mL of appropriate extraction buffer using a vortex and subsequently centrifuged for 5 min at 2000× *g* using a refrigerated centrifuge. For subsequent ELISA analysis, stool sample supernatants (eluates) were used immediately after their preparation.

Blood samples were collected into commercially available serum-separating tubes (Vacutainer, Becton Dickinson, Franklin Lakes, NY, USA). After collection, the blood was allowed to clot at room temperature for 30 min. The clot was removed by centrifugation for 10 min at 2000× *g* using a refrigerated centrifuge. Serum samples were stored at −80 °C immediately after their preparation.

Fecal eluates and serum samples, belonging to samples taken from each dog during standard screening controls, were used to perform a complete biochemical evaluation (for the purposes of the present study, only the values of albumin, cholesterol, and C-reactive protein (CRP—measured on 14 IL dogs) are reported). Then, serum resulting from that used for clinical purposes was used for the evaluation of bacterial LPS (Canine Lipopolysaccharides^®^ ELISA Kit, MyBioSource, San Diego, CA, USA, catalog #MBS2603942), cCK18 (M30 Apoptosense^®^ ELISA Kit, Peviva Inc., West Chester, OH, USA), and citrulline (mass spectrometry—San Marco Laboratories, Padua, Italy). Fecal and serum zonulin concentrations were measured, respectively, using an ELISA kit (Canine Haptoglobin ELISA Kit^®^ (cat.nr. ab137978), Abcam—for serum) and a competitive ELISA assay: Zonulin (feces) EIA (DRG International Inc., Springfield, NJ, USA, catalog number EIA-5418). Commercially available enzyme-linked immunosorbent assay (ELISA) kits were used to measure the different parameters, according to the manufacturers’ protocol. Overflow values and those under the limit of detection for every biomarker were standardized as double and half of the detection limit, respectively [26].

### 2.3. Histopathology

Ten biopsy samples per dog were taken from macroscopically affected areas of the proximal portion of the small intestinal mucosa in the ILG, or random in the CG, fixed in 10% buffered formaldehyde, then embedded in paraffin, and oriented in a sagittal (five specimens) and coronal (five specimens) plane. The histologic examination of H&E-stained sections included the assessment of the histological score [27], evaluated in each biopsy. Villous assessment included height, width, and the h/w ratio evaluation for the villi and villous lymphatic vessels (chyliferous ducts). The means of the values obtained per biopsy were compared between ILG and CG using Student’s *t*-test. Values obtained were compared to the range described in a previous paper and scored (0–3) [1].

### 2.4. Fecal Proteomics

The experimental design of the present proteome analysis was based on the complete sample pooling strategy, as described in previous studies [23,28,29]. Before performing 2DE analysis, fecal samples were treated as described previously; 15 g of the feces pool coming from 13 patients of the ILG (in three cases, it was not possible to obtain a fecal sample to use specifically for proteomics) was resuspended in 22.5 mL of phosphate-buffered saline (PBS) diluted 1:100, containing a protease inhibitor cocktail (Sigma-Aldrich, Saint Louis, MO, USA), and extracted as described by Cerquetella and coworkers [22]. On the final fecal protein samples obtained, the total proteins were determined according to the Bradford method [30]. One milligram of total protein was treated with the 2D Clean-Up Kit (GE-Healthcare Life Sciences, Uppsala, Sweden) and loaded on the two-dimensional electrophoresis (2DE) machine. The analysis was performed in triplicate, in a pH range of 3–10 for the first dimension (Immobiline DryStrip, IPG-strip, length 18 cm; IPGphor isoelectric focusing cell, GE-Healthcare) and 13% SDS-PAGE (Protean II apparatus, Bio-Rad, Hercules, CA, USA) for the second dimension, as described in previous studies [22,29,31]. The stained gels were scanned at 600 dpi and subjected to image analysis using the PDQuest software (Version 7.1.1; Bio-Rad Laboratories), to calculate the isoelectric point (pI), the molecular mass (Mr), and the normalized quantity of each protein spot. The selected spots were manually excised (1 mm in diameter), and the protein was extracted from the gel as described by Shevchenko and coworkers [32], before being subjected to LC–MS/MS analysis for protein identification. The latter was performed as previously described by Cerquetella and coworkers [22]. The MS spectra were extracted and analyzed by MASCOT and SONAR software (www.matrixscience.com; http://hs2.proteome.ca/prowl/knexus.html (accessed on 10 March 2021)). The search parameters were as follows: database, NCBInr; taxonomy, Mammalia; enzyme, trypsin; peptide tolerance, 1.2 Da; MS/MS tolerance, 0.6 Da; allowance of one missed cleavage [22].

### 2.5. Statistical Analysis

The evaluation of the normality of parameters was performed using the Shapiro–Wilk test. Student’s *t*-test was used for comparison of normally distributed data. The Mann–Whitney test was used for the comparison of non-normally distributed data. The differences were considered statistically significant at *p* < 0.05. Statistical data analysis was performed using MedCalc software version 15.8© 1993–2015.

For statistical analysis of fecal proteomics results, a one-way ANOVA online calculator was employed (https://www.statskingdom.com/180Anova1way.html (accessed on 10 March 2021)). Significant differences between means were indicated when *p* < 0.05.

## 3. Results

### 3.1. CIBDAI Score

Clinically the ILG group showed significantly higher CIBDAI scores than the CG (12.31 vs. 1.00; *p* = 0.007) (Table 1).

### 3.2. Intestinal Markers

Ten out of 16 ILG dogs (62.5%) showed albumin values lower than 20 g/L, seven out of 16 (43.7%) showed cholesterol values lower than 92 mg/dL, and 11 of 16 (68.7%) showed CRP values higher than 7.6 mg/L. When comparing the mean values of such markers between ILG and CG, differences were in all cases statistically significant for serum albumin, cholesterol, and CRP (Table 1). Moreover, serum concentrations of bacterial LPS and of cCK18 were different between the two groups, increased in ILG (Table 1). No differences were found for serum citrulline (Table 1). On the contrary, zonulin increased both in serum and in feces in the ILG group (Table 1).

### 3.3. Histopathology

The histological score differed between the two groups (Table 1). Statistically significant differences were also found comparing intestinal villi measurements between ILG and CG, for all evaluations: villous height, width, and h/w ratio, as well as lymphatic vessel (chyliferous duct) height, width, and h/w ratio (Table 1).

### 3.4. Fecal Proteomics

**Figure 1:**
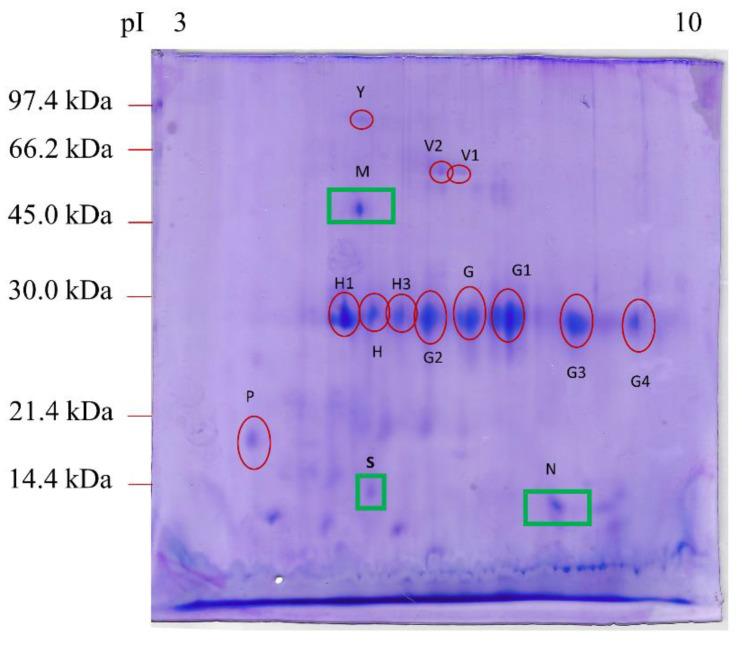
2DE proteomic map of feces from dogs affected by intestinal lymphangiectasia. The protein spots marked with a red circle were expressed in the feces of both healthy dogs (Cerquetella et al., 2021) and dogs with IL. The protein spots marked with a green square were found only in IL-affected CAI feces. The proteins were separated on an immobilized pH 3–10 linear gradient strip and subsequently subjected to 13% SDS-PAGE. The standards were as follows: Bio-Rad low-molecular-weight phosphorylase b, 97.4 kDa; bovine serum albumin, 66.2 kDa; ovalbumin 45.0 kDa; carbonic anhydrase, 31.0 kDa; soybean trypsin inhibitor, 21.5 kDa; lysozyme, 14.4 kDa.

Figure 1 shows the protein expression profile obtained after 2DE in the feces of dogs affected by IL. This protein profile was compared to that obtained from the feces of healthy dogs in a previous study [23].

As shown in Table 2, after the mass spectrometry analysis, we identified 15 main spots, three of which (M, N, and S), corresponding to the Fc fragment of IgG binding protein (Fcgbp), transthyretin (TTR), and proproteinase E, respectively, were present in the feces of dogs with lymphangiectasia but not in healthy dogs [23].

No significant differences were found between healthy dogs and IL dogs concerning the number of spots: spot Y (albumin isoform X1), spots V1 and V2 (alkaline phosphatase), spot H (chymotrypsin-C-like), spot H1 (elastase-3B), spot H3 (immunoglobulin kappa light chain), spots G and G1 (immunoglobulin λ-1 light chain), spots G2, G3, and G4 (corresponding to forms of immunoglobulin λ-light chain VLJ region), and spot P (deleted in malignant brain tumors 1 protein isoform X1).

Lastly, in dogs of the present study, we did not find some spots (attributed to specific proteins), previously detected in healthy dogs [23]: a serum albumin isoform X1, other than our spot Y, a nuclear pore membrane glycoprotein-210, and a cytosol aminopeptidase.

## 4. Discussion

As expected [2,6,33], in the ILG, serum levels of albumin, cholesterols and CRP differed from the CG; more precisely, albumin and cholesterol were reduced in IL, while CRP was increased compared to CG.

Low blood albumin level is the most consistent finding in lymphangiectasia. Usually, both albumin and globulin are lost from the intestines in protein-losing enteropathies, especially where there is a marked villous atrophy. The decreased albumin production could be due to a hepatic insufficiency, while the increased albumin loss could be due to a protein-losing nephropathy, as well as acute or chronic blood loss, and it should be excluded diagnostically. Furthermore, albumin is a negative acute-phase protein that is decreased in inflammatory diseases. Cholesterol level is also decreased in protein-losing enteropathies and in malabsorption. It is part of the lymph fluid that gets lost. CRP is a positive acute-phase protein (major responder in dogs), and it is useful for diagnosis and monitoring recovery from the acute phase of disease [7,34].

Bacterial LPS can be considered an indirect indicator of bacterial translocation, and it has some proinflammatory activity. Indeed, when LPS is recognized by Toll-like receptor 4, it is activated, leading to the synthesis of proinflammatory mediators (cytokines and chemokines) [35]. LPS was also shown to be able to induce lung edema in an experimental context [36], and its absorption seems to be linked to fat intake [37]. Interestingly, its increase in our sets of IL patients may suggest a loss of mucosal intestinal barrier integrity that is expected, as also shown by histopathology.

Similarly, cCK18 may represent an indirect way to evaluate apoptosis levels in enterocyte population. Indeed, the ratio between the caspase-cleaved fragment of cytokeratin 18 and total K18 in serum has been defined as the apoptotic index [38]. The retrieval of a higher concentration of that molecule in ILG of the present study, compared to CG, could suggest a more serious mucosal damage, correlating with LPS serological levels and histopathological findings.

Citrulline is considered an indicator of enterocyte mass and intestinal absorption in human medicine, but it has not been associated with treatment outcome in dogs with chronic enteropathy [39]; despite the absence of statistical significance, it is noteworthy that, in our samples, it resulted increased compared to controls.

Lastly, zonulin, which is a tight junction protein linked to intestinal barrier integrity, was found to be increased in intestinal biopsies of IBD dogs treated with probiotics (plus standard therapy, including prednisone), compared to controls [40], when evaluated by immunohistochemistry in intestinal biopsies; however, it also decreased after therapy (prednisone and diet) in canine IBD, as well as in intestinal biopsies [41]. We aimed to measure such a molecule in the serum and feces of IL dogs to indirectly evaluate mucosal integrity. We found significantly increased levels of zonulin in ILG compared to CG, in both serum and feces, suggesting that it may be increased in such samples from diseased patients following mucosal damage. If the apoptotic index of enterocytes represents an important parameter for evaluating the integrity of the mucosal barrier, the tightness of the intercellular junctions between the enterocytes themselves is even more important. The tight junctions (TJs) surround the apical portion of the enterocytes and regulate the paracellular permeability of solutes. TJs are multiprotein complexes, and the intracellular domain of these proteins interacts with cytosolic proteins, the zonula occludens (ZO) proteins, which anchor the transmembrane proteins to the actin cytoskeleton. The interaction between the tight junctions and the actin cytoskeleton is fundamental to maintain the tight junction structure, allowing regulation of the paracellular pathway [42]. One of the modulators of these TJs is zonula occludens toxin (Zot) [43,44]. Zonulin, a 47 kDa protein, represents an endogenous analog of Zot, and it is related to a family of proteins and a precursor of haptoglobin 2 (HP2) [45,46], which is overexpressed in pathological conditions that alter intestinal permeability [47]. In humans, the expression of zonulin has been related to several pathologies and linked as a biomarker of gut permeability [48]. Studies have shown that zonulin has been implicated in many intestinal and metabolic pathologies; in all of these conditions, an increase in the permeability of the intestinal barrier generates an early disassembly of the enterocytes and a release of zonulin which passes freely into the serum and directly into the intestinal lumen [12]. Following the release of zonulin, the intestine shows increased permeability and disassembly of ZO-1 from the tight junction complex [12]. In particular, the increase in expression of CXCR3 receptor on the apical surface of enterocytes with subsequent MyD88 (a key adapter molecule in the TLR signaling pathway)-dependent zonulin release has been observed [12]. In these conditions, extra zonulin is also released from MyD88-dependent macrophages, as described in [12]. In addition, different studies suggest that, in addition to regulating the intestinal epithelium, zonulin participates in the regulation of extraintestinal epithelia and the vascular endothelium, which also contain tight junctions. In this context, zonulin is of importance in the regulation of permeability across all endothelial and epithelial surfaces, e.g., brain, intestinal epithelium, vascular and lymphatic vessels, and lung tissue. Thus, fecal zonulin may be more associated with intestinal permeability, since secretion of zonulin from the intestinal barrier may leak into the lumen, whereas serum zonulin originates from several different tissues. These considerations are also in accordance with Sturgeons et al.’s [12] explanation that there is no correlation between serum and fecal levels of zonulin. Although HP2 also represents an acute-phase protein in dogs [49], its increase in the serum of dogs of the ILG group might be normal since they have an enteropathy. In contrast, from our results, it can be assumed that the increase in fecal zonulin relative to serum zonulin in dogs with IL may be related to the histopathological lesion of intestinal biopsies and villous/lactal dilation, and there may be a difference with the observed value in CG dogs, indicating a direct involvement of this molecule in the condition of “leaky gut” [48].

With regard to fecal proteome evaluation, three proteins were found in IL but not in healthy dogs [23]. Very interesting is the presence of transthyretin (TTR, spot N). TTR is a tetrameric plasma protein synthesized mainly in the liver, but also in the brain and in the pancreas. The main function of this protein is to transport retinol (vitamin A) and the thyroid hormones triiodothyronine (T3) and thyroxine (T4) in plasma [50]. Amyloidogenic TTR mutations can destabilize the tetramer, leading to the monomer dissociation; TTR monomers can aggregate to form beta-structured fibrils that give rise to extracellular amyloid deposits, a condition known as transthyretin amyloidosis that may lead to progressive damage of the autonomic nervous system. In human medicine, it is reported that gastrointestinal disturbances may arise from hereditary transthyretin amyloidosis, since the impairment of the enteric nervous system can lead to a reduced GI motility [51]. Our fecal proteomic analysis also highlighted the presence of Fcgbp. In humans, Fcgbp is expressed in the mucus granule of colon goblet cells, and it can bind to the Fc part of IgG antibodies but not to IgM or IgA [52]. However, the presence of a sequence with the repetitive Von Willebrand factor (vWF) type D domain suggests other functions for the Fcgbp. Indeed, it is known that the vWF type D domain controls and mediates N-terminal oligomerization and is found in oligomer-forming proteins (e.g., mucins and the von Willebrand factor). Johansson and coworkers [52] found that Fcgbp with its repetitive vWF type D domain is covalently attached to the Muc2 mucin, and this suggests that Fcgbp may play a role in immune protection and inflammation in human intestines [53]. This is consistent with a previous study by Kobayashi and coworkers [54], where it was found that, in the intestinal goblet cells of patients affected by ulcerative colitis and Crohn’s disease, Fcgbp is highly expressed. The third protein was proproteinase E, which is a pancreatic precursor (zymogen) of an enzyme belonging to the pancreatic elastase/proteinase E subfamily and, thus, considered a chymotrypsin-like serine endopeptidase [55,56]. In consideration of this, it is easy to explain its presence in the feces of our patients, although it is unfortunately not simple to elucidate why it was not detected in a previous study in the feces of healthy dogs [23]. It can, however, be speculated that the likely increased intestinal transit time in diseased dogs may explain this result. Another explanation could be the greater quantity of undigested lipids and proteins in the feces of IL dogs, which would stimulate a greater production of the whole machinery complex of enterocyte brush border proteins [57]. In the IL condition, the normal catabolism of some large intestinal brush border proteins is increased, and it is demonstrated that the surface of intestinal absorptive cells is being constantly remodeled; in these conditions, certain surface enzymes are in part removed from the membrane by the action of pancreatic proteases, principally elastase [58].

Moreover, it is worth noting that none of the three aforementioned proteins were previously found in a fecal proteome evaluation performed on dogs suffering from food-responsive diarrhea [22]; however, in two cases (spots M and N), similar proteins were found in the feces of healthy cats (IgGFc-binding protein, and transthyretin precursor) [23], which prevents any specific consideration.

Lastly, although in the absence of statistical significance, it should be reported that, in the feces of our patients, we found levels of serum albumin isoform X1 lower than those reported in healthy dogs [23]. This is expected, as we also found that patients with IL have lower serum albumin levels than CG, whereas it was also expected for us to find such proteins in the feces (if IL is considered a PLE); however, our findings are in contrast with this assumption. Further studies are needed to investigate and clarify this apparent contradiction and the reason(s) behind its absence.

The fact that zonulin was not identified by the proteomic study, while it was conversely found in feces with a specific test, is due to the lower sensitivity of 2DE with respect to specific tests based on the use of specific antibodies. The stool biomarker candidates of ILG that were found in this study can be clinically applied after validation by studies that use target-based high-throughput methods, such as ELISA and selected reaction monitoring (SRM). In particular, zonulin (HP2) observed by ELISA but not clearly evidenced by fecal proteomics could be evaluated using a different proteomic approach. Therefore, it is quite important to validate our preliminary results with larger cohorts.

Proteomics is very useful to evidence high-abundance proteins; however, there are proteins present at very low concentrations ranging from ng/mL to pg/mL, which are difficult to determine through the proteomic analysis carried out with 2DE, since they are masked by the high-concentration proteins [59]. These low-concentration proteins often leak or are shed from tissues (including diseased cells/tissues), whereas they may also represent interleukins, cytokines, or growth factors [60,61]. These low-abundance proteins potentially hold critical information regarding the health and disease status of any individual [62].

## 5. Conclusions

In the present study, serum albumin and cholesterol levels resulted decreased in dogs suffering from IL when compared to patients without IL. Conversely, serum concentrations of CRP, bacterial LPS, cCK18, and zonulin were higher in IL dogs, as also shown for fecal zonulin, necessitating a further investigation of their possible application as markers of IL in dogs. No statistically significant differences were found for serum citrulline. Clinical score and histopathology also showed differences between ILG and CG, with both increased in IL. Interesting alterations were also found, similarly to a previous study [1], in villi height and width, with the former reduced and the latter increased in ILG compared to CG. Fecal proteomics allowed the retrieval of three proteins in IL that were not previously found in healthy dogs [23] or in dogs suffering from food-responsive diarrhea [22]. The present data need to be confirmed and enriched with future findings in order for a better understanding; however, these results represent important steps toward the possible identification of new markers of disease.

## Figures and Tables

**Table 1:** Comparison between ILG and CG with regard to clinical score, intestinal markers, and histological evaluations.

	Case (ILG) Mean Value ± DS	Control (CG) Mean Value ± DS	* p
CIBDAI	12.31 ± 2.75	1 ± 0.81	0.007
Serum			
Albumin	17.32 ± 4.23	32.14 ± 3.84	<0.0001
Cholesterol	110.75 ± 39.89	212.28 ± 18.81	<0.0001
CRP	26.83 ± 16.98	1.21 ± 0.6	0.0009
LPS	0.52 ± 0.30	0.1 ± 0.05	0.0001
cCK18	365.81 ± 104.92	148.71 ± 74.68	0.0001
Citrulline	4.56 ± 1.59	2.9 ± 3.10	0.14
Zonulin	77.27 ± 32.38	38.91 ± 19.10	0.0181
Feces			
Zonulin	523.20 ± 156.42	141.56 ± 72.67	<0.0001
Biopsies			
Histological score	13.5 ± 2.25	1.85 ± 1.21	<0.0001
Villous height	623.31 ± 17.14	809.57 ± 26.78	<0.0001
Villous width	277.25 ± 11.03	151.42 ± 11.68	<0.0001
Villous h/w ratio	2.25 ± 0.11	5.37 ± 0.45	<0.0001
Lymphatic vessels (chyliferous ducts) height	551.18 ± 24.03	658.71 ± 33.50	<0.0001
Lymphatic vessels (chyliferous ducts) width	107.50 ± 42.68	23.85 ±2.47	<0.0001
Lymphatic vessels (chyliferous ducts) h/w ratio	5.95 ± 2.32	27.79 ± 3.56	<0.0001

* Statistically significant *p* < 0.05.

**Table 2:** Fecal proteins from dogs affected by IL compared with the fecal proteins from healthy dogs [23]. The proteins were identified by LC–MS/MS followed by MASCOT and SONAR software analysis [www.matrixscience.com (accessed on 10 March 2021); http://hs2.proteome.ca/prowl/knexus.html (accessed on 10 March 2021)].

SPOT ID ^a^	Protein Name ^b^	Score ^c^	Mr (kDa)/pI ^d^	Mr (kDa)/pI ^e^	Sequence	NQ ^e^ (×10^3^) Healthy *	NQ (×10^3^) IL
Y	Serum albumin isoform X1 (*Canis lupus familiaris*)	56	68.6/5.51	72 ± 4.6/5.3 ± 0.06	LVAAAQAALV	115 ± 47	71 ± 42
V1	Alkaline phosphatase (*Canis lupus familiaris*)	125	68.6/6.47	59 ± 1.3/6.6 ± 0.23	ANYQTIGVSAAAR	174 ± 108	47 ± 26
V2	Alkaline phosphatase (*Canis lupus familiaris*)	117	48.3/6.15	58 ± 1.6/6.6 ± 0.15	ANYQTIGVSAAAR	148 ± 51	35 ± 18
H	Chymotrypsin-C-like (*Canis lupus dingo*)	49	29.1/5.33	29 ± 1.0/5.6 ± 0.11	LAEPVQLSDTIK	290 ± 93	264 ± 27
H1	Elastase-3B, proteinase E (*Canis lupus familiaris*)	40	28.8/5.27	29 ± 0.8/5.2 ± 0.11	VSAFNDWIEEVMSSH	585 ± 139	616 ± 351
H3	Immunoglobulin kappa light chain (*Felis catus*)	41	26.7/6.10	29 ± 1.1/6.3 ± 0.21	FSGSGSGTDFTLR	374 ± 248	214 ± 32
G	Immunoglobulin λ-1 light chain (*Canis lupus familiaris*)	34	25.2/6.88	29 ± 0.9/7.1 ± 0.5	KGTHVTVLGQPK	644 ± 327	570 ± 59
G1	Immunoglobulin λ-1 light chain (*Felis catus*)	39	27.8/8.17	29 ± 1.2/7.6 ± 0.6	QSNNKYAASSYL	555 ± 204	232 ± 142
G2	Immunoglobulin λ-light chain VLJ region (*Homo sapiens*)	42	29.0/8.14	29 ± 1.0/6.6 ± 0.3	EFGGGTKLTVLGQPK	642 ± 439	321 ± 92
G3	Immunoglobulin λ-light chain VLJ region (*Homo sapiens*)	30	29.0/8.14	27 ± 1.2/8.4 ± 0.5	EFGGGTKLTVLGQPK	395 ± 85	483 ± 64
G4	Immunoglobulin λ-light chain VLJ region (*Homo sapiens*)	40	29.0/8.14	27.7/8.9	QSNNKYAASSYL	285 ± 17	334 ± 271
M	Fc fragment of IgG binding protein (*Homo sapiens*)	23	572/5.1	49.1 ± 2.2/5.3 ± 0.06	VLVENEHR	n.d.	148 ± 25
N	Transthyretin (*Canis lupus familiaris*)	31	15.8/6.4	14.2 ± 1.2/7.8 ± 0.1	GSPAVNVAVK	n.d.	62 ± 58
P	Deleted in malignant brain tumors 1 protein isoform X1 (*Canis lupus dingo*)	55	26/5.2	19.8 ± 0.15/4.2 ± 0.3	FGQGSGPIVLDDVR	196.5 ± 53.2	132 ± 39
S	Proproteinase E (*Bos taurus*)	43	27.3/5.1	14 ± 3.4/5.8 ± 0	LYTGGPLPDK	n.d.	103 ± 78
	^§^ * Serum albumin isoform X1 (*Canis lupus familiaris*)		68.6/5.51	63 ± 3.0/5.8 ± 0.15	ADFAEISK	79 ± 13	n.d.
	^§^ * Nuclear pore membrane glycoprotein-210 (*Canis lupus familiaris*)		192.4/6.30	19.6 ± 1.5/5.8 ± 0.14	TALLVTASISGSHAPR	227 ± 69	n.d.
	^§^ * Cytosol aminopeptidase (*Canis lupus familiaris*)		56.2/8.03	21.0 ± 1.3/5.7 ± 0.07	EILNISGPPLK	125 ± 32	n.d.

NQ: normalized quantity. n.d.: not detected. ^a^ Assigned spot ID as indicated in Figure 1. ^b^ MASCOT results (SwissProt and NCBInr databases). ^c^ MASCOT score reported. ^d^ From SwissProt and NCBInr databases. ^e^ Experimental values were calculated from the 2DE maps by the PDQuest software (±standard deviation). * Normalized quantities from healthy subjects were obtained from a previous study from the same research group [23]; ^§^ spots absent in IL but present in clinically healthy dogs.

## Data Availability

Data are contained within the article. Some data included in the present manuscript have already been presented as abstract at the Joint Congress of the European College of Veterinary Pathologists, the European Society of Veterinary Pathology, the European College of Veterinary Clinical Pathology and the European Society of Veterinary Clinical Pathology, 2019 [*“Evaluation of some potential new serological and fecal markers in canine lymphangiectasia: correlation with mucosal morphology and histological score”*, Rossi, 2020].
